# Regulatory Elements Outside Established *Pou5f1* Gene Boundaries Are Required for Multilineage Differentiation of Embryonic Stem Cells

**DOI:** 10.3390/ijms242015434

**Published:** 2023-10-21

**Authors:** Veronika V. Ermakova, Nikita P. Fokin, Nikolay D. Aksenov, Evgeny I. Bakhmet, Ekaterina V. Aleksandrova, Andrey A. Kuzmin, Alexey N. Tomilin

**Affiliations:** Institute of Cytology, Russian Academy of Sciences, 194064 St.-Petersburg, Russia; v.ermakova@incras.ru (V.V.E.); fokinnikspb@gmail.com (N.P.F.); aksenovn@gmail.com (N.D.A.); aleksandrova.biotech@gmaii.com (E.V.A.); a.kuzmin@incras.ru (A.A.K.)

**Keywords:** *Pou5f1*, embryonic stem cells (ESCs), pluripotency, differentiation, naïve and primed states of pluripotency, regulation of gene expression

## Abstract

The transcription factor Oct4 can rightfully be considered a pivotal element in maintaining pluripotency. In addition, its ability to function as a pioneer factor enables the reprogramming of somatic cells back into a pluripotent state. To better understand the regulation of the Oct4-encoding gene (*Pou5f1*), the main genetic elements that regulate its expression in different states of pluripotency ought to be identified. While some elements have been well characterized for their ability to drive *Pou5f1* expression, others have yet to be determined. In this work, we show that translocation of the *Pou5f1* gene fragment purported to span all essential *cis*-elements, including the well-known distal and proximal enhancers (DE and PE), into the *Rosa26* locus impairs the self-renewal of mouse embryonic stem cells (ESCs) in the naïve pluripotency state, as well as their further advancement through the formative and primed pluripotency states, inducing overall differentiation failure. These results suggest that regulatory elements located outside the previously determined *Pou5f1* boundaries are critical for the proper spatiotemporal regulation of this gene during development, indicating the need for their better characterization.

## 1. Introduction

Oct4 is a POU domain transcription factor encoded by the *Pou5f1* gene, which is indispensable for maintaining the pluripotent state in embryonic stem cells (ESCs) [1]. Although Oct4 functions and mechanisms of its action as a transcription factor have been extensively studied [2,3], information about the regulation of the *Pou5f1* gene remains limited. On the other hand, the gene ought to be precisely regulated because rather subtle variations in Oct4 protein levels have been shown to exert quite diverse effects on the fate of pluripotent cells [4,5]. Additionally, some *Pou5f1* gene elements are important in non-pluripotent cells, for example, in the context of autoimmune diseases [6]. And while it is widely accepted that *Pou5f1*, particularly via the expression of the Oct4 protein isoform A, plays important roles exclusively in pluripotent stem cells and cells of germline lineage [1,7], new data challenging this paradigm have been emerging, further emphasizing the necessity of studying *Pou5f1* regulation [8,9,10].

In mice, *Pou5f1* is regulated via a TATA-less promoter and two upstream enhancers, the distal (DE) and proximal (PE) ones, which act in a stage-dependent manner [11]. The most comprehensive examination of the functioning of these enhancers in naïve vs. primed mouse ESCs was performed by Choi et al., who demonstrated that DE directs *Pou5f1* transcription in naïve ESCs, which corresponds to the early preimplantation epiblast cells. Conversely, in the primed state corresponding to the post-implantation epiblast, control over *Pou5f1* expression shifts to the proximal enhancer (PE) [12,13,14]. Similarly, in humans, *POU5F1* expression is thought to be governed primarily by three enhancers: the orthologs of the mouse DE and PE, as well as by an intronic enhancer responsible, along with DE, for maintaining *POU5F1* expression in the naïve state [15]. Furthermore, high-throughput genome engineering approaches have been deployed to identify other non-canonical enhancers that can affect *POU5F1* expression. Diao et al. developed the method called CREST-seq, which helped to identify 45 *cis*-regulatory elements of the *POU5F1* gene located within the 2-Mbp *POU5F1* locus [16]. Although these regulatory elements showed an impact on *POU5F1* expression in ESCs, the consequences of their perturbations in vivo are still to be evaluated. Also, the collective contribution of these elements remains unclear.

Here, we sought to explore putative regulatory elements located outside the established *cis*-regulatory elements of *Pou5f1* in the context of pluripotency transition from naïve to primed states. Using a reverse genetic approach, we transferred the *Pou5f1* gene fragment, previously shown to span essential functional elements, into ectopic chromosome context and found dramatic changes in Oct4 regulation along with severely compromised ESC differentiation capacity.

## 2. Results

**Figure 1:**
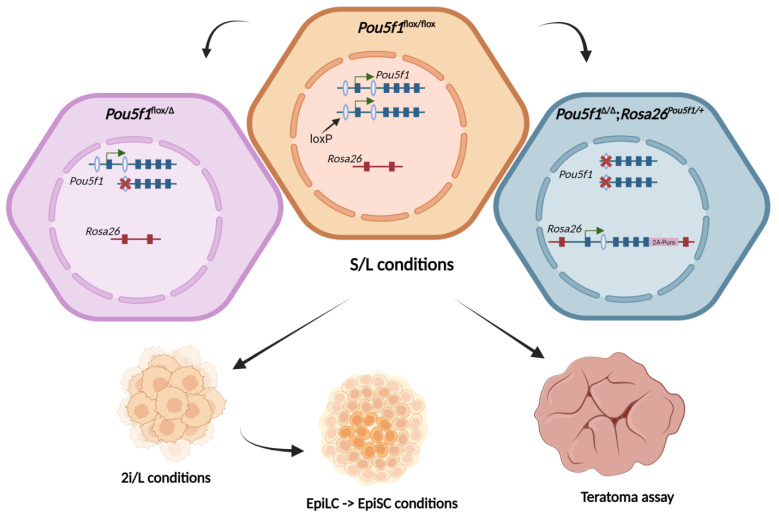
Schematic outline of experiments. Control *Pou5f1^flox/∆^* cells were established in the present work from the parental *Pou5f1^flox/flox^* ESC line [24] by partial Cre-recombination. Experimental *Pou5f1^∆/∆^;Rosa26^Pou5f1/+^* ESC line was established earlier from the same parental cells [17]. Cells were cultured in the S/L media and injected into immune-incompetent mice to assess their differentiation potential via teratoma formation assay. Also, the cells were examined for their ability to perform in different states of pluripotency—naïve (as ESCs in the 2i/L), formative (as EpiLCs), and primed (as EpiSCs). White ellipses with blue outline represent LoxP sites, exons are depicted as navy blue (*Pou5f1*) or dark red (*Rosa26*) rectangles. Created with BioRender.com.

In our previous report, we were able to rescue the self-renewal of mouse null-Oct4 ESCs using the 9.8 kb fragment of the *Pou5f1* gene, which spanned the previously mapped DE and PE, by inserting this fragment into one of the *Rosa26* alleles (Figure S5). The derived ESCs, referred to as *Pou5f1^∆/∆^*;*Rosa26^Pou5f1/+^*, showed normal proliferation and the expression of the main pluripotency markers (Oct4, Nanog, Klf4) when cultured in the presence of serum and leukemia inhibitory factor (LIF), hereafter designated as S/L conditions [17]. Accordingly, previous data reported that the monoallelic expression of *Pou5f1* was sufficient to maintain the pluripotent state of *Pou5f1^+/–^* ESCs, which was, surprisingly, even more robust than that of its bi-allelic counterparts [18]. In this paper, we generated an analogous monoallelic ESC line, *Pou5f1^flox/Δ^* (see Section 4), and set out to compare it to the parental *Pou5f1^flox/flox^* and *Pou5f1^∆/∆^*;*Rosa26^Pou5f1/+^* cells for the ability to self-renew in different states of pluripotency, i.e., as naïve ESCs, epiblast-like cells (EpiLCs), and epiblast stem cells (EpiSCs) [19,20,21,22,23], as well as for the ability to terminally differentiate in teratoma formation assay (as outlined in Figure 1).

**Figure 2:**
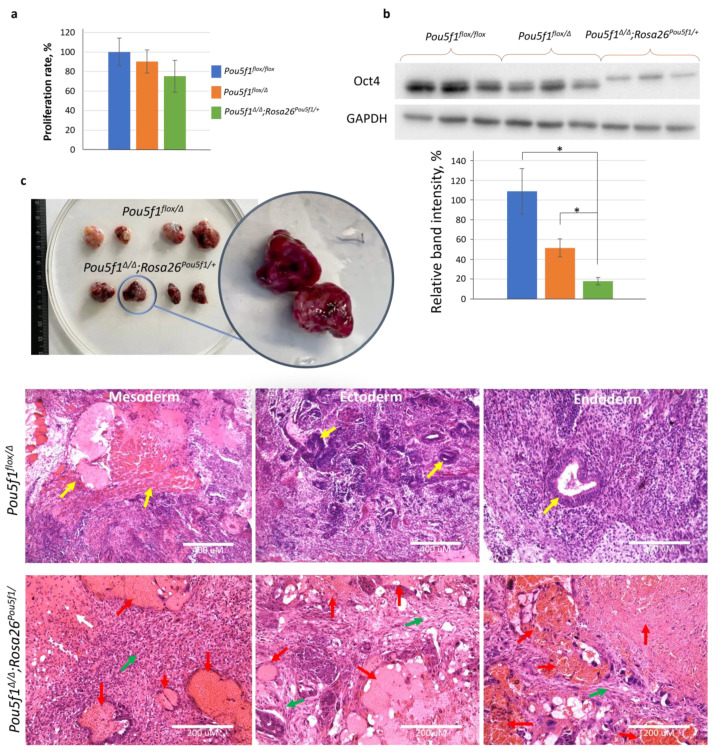
Assessment of ESC properties under the S/L conditions. (**a**) ESC lines of the three indicated genotypes demonstrated non-significant differences in proliferation rates; genotype designation is as follows: blue—*Pou5f1^flox/flox^,* orange—*Pou5f1^flox/Δ^*, green—*Pou5f1*^∆/∆^;*Rosa26^Pou5f1/+^*. (**b**) Expression level of Oct4 protein correlates with *Pou5f1* copy number, shown via immunoblot analysis using Oct4 antibodies; the shift of the Oct4 molecular weight in *Pou5f1*^∆/∆^;*Rosa26^Pou5f1/+^* cells is explained in the text; GAPDH antibodies were used for normalization; and the Oct4 levels in *Pou5f1^flox/flox^* ESCs was taken as 100%. The *Y* axis represents the mean band intensity (the genotypes are color-coded as in Figure 2a). Whiskers indicate standard error of mean (SEM), *n* = 4 (biological replicates) for proliferation and apoptosis experiments, *n* = 3 for protein measurements, * *p* < 0.05. (**c**) Cell lines showed varying differentiation potential in vivo in teratoma formation assay. Teratomas formed using *Pou5f1*^∆/∆^;*Rosa26^Pou5f1/+^* ESCs were mainly composed of blood-filled cavities along with some cartilage-like (white arrow) and connective tissues (green arrows), both of mesodermal origin. Yellow arrows indicate germ layer derivatives formed by *Pou5f1^flox/Δ^* ESCs—mesoderm (muscle), ectoderm (neuronal tissue), and endoderm (respiratory epithelium). White arrow indicates cartilage, green and red arrows indicate mesenchymal connective tissues and blood-filled cavities, respectively.

To characterize the ESC lines under the S/L conditions, we compared their proliferation rates and evaluated the Oct4 levels using a Western blot. The three cell lines exhibited similar proliferation characteristics (Figure 2a), while the Oct4 protein level in *Pou5f1^flox/Δ^* and *Pou5f1^∆/∆^;Rosa26^Pou5f1^* ESCs was, respectively, 50% and <25% of that in *Pou5f1^flox/flox^* control cells (Figure 2b). The molecular weight shift of Oct4 species in *Pou5f1^∆/∆^;Rosa26^Pou5f1/+^* cells was apparently due to a portion of the 2A polypeptide remaining covalently linked to the Oct4 C-terminus after self-cleavage of the Oct4-2A-Puro fusion protein. As proof of this statement, the same molecular weight Oct4 species was generated from the *Pou5f1^flox−2A-Puro/∆^* allele (Figure S1). Teratomas formed using *Pou5f1^flox/Δ^* ESCs contained derivatives of all three germ layers, whereas *Pou5f1^∆/∆^;Rosa26^Pou5f1/+^* ESCs gave rise to tumors with drastically different appearances, as already evident prior to histological analysis (Figure 2c). This analysis showed that blood-filled cavities comprised most of *Pou5f1^∆/∆^;Rosa26^Pou5f1/+^* tumor volume. Solid tissue from these teratomas did not contain germ layer derivatives readily present in *Pou5f1^flox/Δ^* sections but instead cartilage-like and mesenchymal connective tissues, providing a clue about *Pou5f1^∆/∆^;Rosa26^Pou5f1/+^* ESC differentiation bias towards the mesoderm lineage (Figure 2c).

**Figure 3:**
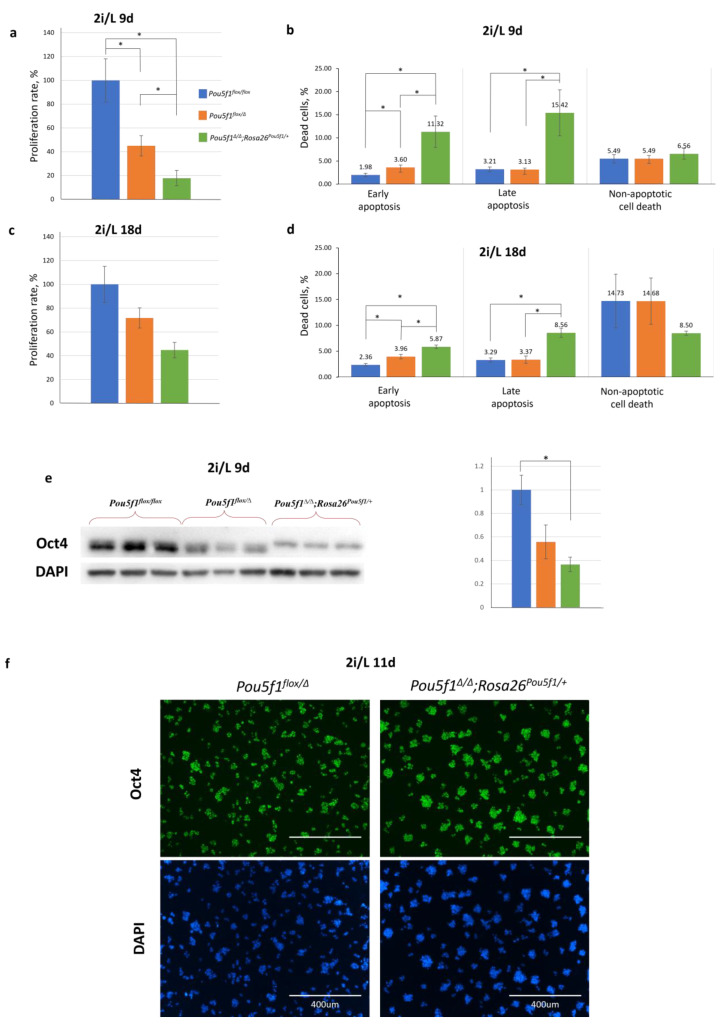
Assessment of ESC properties under the 2i/L (naïve) conditions. (**a**,**b**) As compared to the control *Pou5f1^flox/flox^* ESCs, proliferation of both *Pou5f1^flox/Δ^* and *Pou5f1*^∆/∆^;*Rosa26^Pou5f1/+^* ESCs significantly decreased by day 9 of culturing under the 2i/L conditions, while the latter cells additionally showed an increase in apoptosis. (**c**,**d**) Proliferation of *Pou5f1*^∆/∆^;*Rosa26^Pou5f1/+^* cells recovered approximately twofold, while apoptosis declined about twofold after 18 days of culturing in 2i/L media. (**e**) Western blot analysis (left) and assay quantification of Oct4 protein (right) based on this in the ESCs cultured under the 2i/L conditions for 9 days; GAPDH was used as a reference protein (**f**) Immunocytochemical staining of Oct4 protein was performed on ESCs cultured in 2i/L medium for 11 days. (**a**,**c**) *Y* axis represents the mean proliferation rate of ESC (the Oct4 level in *Pou5f1^flox/flox^* ESCs was taken as 100%). (**b**,**d**) *Y* axis represents the mean dead cells, %. (**e**) *Y* axis represents the mean band intensity. The genotypes are color-labeled as in Figure 2a. Whiskers indicate standard error of the mean (SEM), *n* = 4 (biological replicates) for proliferation and apoptosis experiments, *n* = 3 for protein measurements, * *p* < 0.05.

Contrary to the S/L medium, culturing in a serum-free medium supplemented using GSK/MAPK inhibitors (2i) and LIF, referred hereafter to as 2i/L medium, for 9 days resulted in a proliferation decrease in the *Pou5f1^flox/Δ^* and especially, in *Pou5f1^∆/∆^;Rosa26^Pou5f1/+^* ESCs, as compared to the control *Pou5f1^flox/flox^* ESCs (Figure 3a). In addition, the *Pou5f1^∆/∆^;Rosa26^Pou5f1/+^* ESCs demonstrated a 3- to 5-fold increase in apoptosis compared to the other two lines (Figure 3b). At the same time, the non-apoptotic cell death rate was approximately the same in all three cell lines. Interestingly, after an additional 9 days (18 days in total) of culturing under 2i/L conditions, the proliferation rate of *Pou5f1^∆/∆^;Rosa26^Pou5f1/+^* ESCs increased twofold (Figure 3c), while apoptotic processes reduced almost twofold (Figure 3d), indicating the progressive adaptation of these cells to the 2i/L conditions. Contrary to the S/L conditions (Figure 2b), 2i/L conditions featured Oct4 levels in *Pou5f1^∆/∆^;Rosa26^Pou5f1/+^* ESCs, nearly reaching that in *Pou5f1^flox/Δ^* cells, i.e., 50% of the bi-allelic expression level (Figure 3e,f).

**Figure 4:**
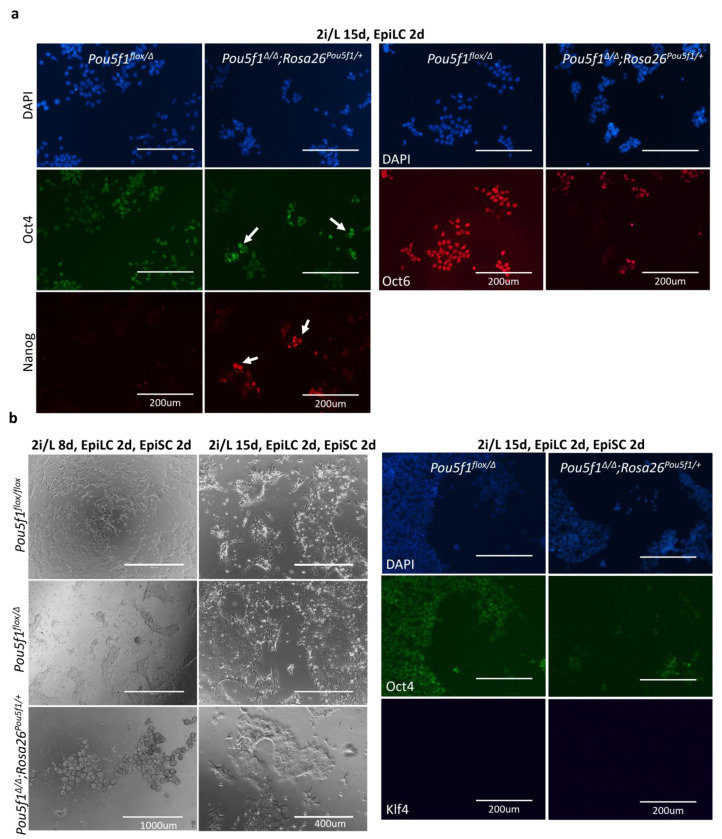
Assessment of cell properties in formative and primed pluripotency states. (**a**) *Pou5f1^∆/∆^;Rosa26^Pou5f1/+^* ESCs cultured as indicated above the panels (supposedly reaching the formative state) incompletely down-regulated Nanog, fail to set Oct6 expression and show mosaic Oct4 pattern, demonstrated by immunostaining; white arrows point to cells with elevated Oct4 or Nanog levels. (**b**) Two days after subsequent culturing under EpiSC conditions, promoting the primed state, *Pou5f1^∆/∆^;Rosa26^Pou5f1/+^* cells are able to attach to the fibronectin-coated surface if cultured under the 2i/L conditions for an extended period (15 days in total) prior to switching to EpiLC/EpiSC conditions; however, these cells still fail to maintain Oct4, as opposed to the *Pou5f1^flox/Δ^* counterparts. Lack of Klf4 staining indicates the exit from the naïve state.

As terminal differentiation of ESCs proceeds through formative and primed pluripotency, we took a closer look at these intermediate states, featured by the cultured epiblast-like cells (EpiLCs) and epiblast stem cells (EpiSCs), respectively [21,22,23]. In the EpiLCs, the differences between the *Pou5f1^∆/∆^;Rosa26^Pou5f1/+^* and *Pou5f1^flox/Δ^* ESCs, observed under the 2i/L conditions, further increased. Expression of the pluripotency state markers Oct4, Nanog, and Oct6 was notably perturbed in *Pou5f1^∆/∆^;Rosa26^Pou5f1/+^* cells (Figure 4a). Nanog expression, which is normally suppressed in the EpiLC state, was retained in a significant fraction of *Pou5f1^∆/∆^;Rosa26^Pou5f1/+^*cells, while the Oct4 expression showed a heterogenous expression pattern, with some cells retaining high levels and the others showing decreased levels of the Oct4 protein. Oct6, in turn, was expressed only in a minor subset of *Pou5f1^∆/∆^;Rosa26^Pou5f1/+^* cells, compared to the control *Pou5f1^flox/Δ^* cells (Figure 4a).

When we proceeded further to EpiSC differentiation, *Pou5f1^∆/∆^;Rosa26^Pou5f1/+^* cells progressively lost their ability to attach to the fibronectin-coated surface, completely changing their morphology. Interestingly, when culturing under the 2i/L conditions was extended to 15 days prior to the primed state induction, differentiated *Pou5f1^∆/∆^;Rosa26^Pou5f1/+^* cells were notably better at attaching to the fibronectin-coated surface, although they still showed deeply perturbed expressions of the EpiSC markers Oct4 and Oct6 (Figure 4b and Figure S3). At the same time, these cells were negative for Klf4 (Figure 4b), indicating their exit from a naïve state. In sum, the obtained data suggest that pluripotency continuum is severely perturbed in *Pou5f1^∆/∆^;Rosa26^Pou5f1/+^* cells. These cells can exit the naïve state; however, they fail (1) to properly maintain the Oct4 levels in formative and primed states, (2) to down-regulate Nanog in the formative state, and (3) to fully establish formative and primed states of pluripotency.

To rule out the possibility of an unspecific impact of the *Rosa26* locus *cis*-regulatory elements on *Pou5f1* transgene expression within the *Rosa26^Pou5f1^* allele, we inserted a cassette expressing tdTomato under the CAG promoter control into the same locus position; we then compared the tdTomato intensity under S/L and 2i/L conditions. We did not observe any increase in the tdTomato signal upon switching from the S/L to 2i/L conditions (Figure S4), supporting the conclusion that the observed differentiation defects of *Pou5f1^∆/∆^;Rosa26^Pou5f1/+^* ESCs occur because of some essential *cis*-regulatory elements missing in the 9.8 kb *Pou5f1* transgene, rather than because of those elements within *Rosa26* modulating the transcriptional activity of this transgene.

## 3. Discussion

In the present work, we evaluated the consequences of the translocation of the *Pou5f1* gene fragment spanning *cis*-regulatory elements, previously defined as directing proper spatiotemporal *Pou5f1* expression during mouse ontogeny—the DE and PE [11]—in an ectopic chromosomal context, as featured by the *Pou5f1^∆/∆^*;*Rosa26^Pou5f1/+^* ESCs. We found that these ESCs resemble those with monoallelic *Pou5f1* expression within a native chromosomal context (*Pou5f1^flox/∆^*) in terms of their self-renewal under S/L conditions; however, these ESCs showed severely compromised differentiation capacity in teratoma assay with a significant bias towards mesoderm. Accordingly, *Pou5f1^∆/∆^;Rosa26^Pou5f1/+^* cells showed decreased proliferation and increased apoptosis rates. When we proceeded to differentiation, these cells failed to properly maintain the Oct4 levels in the formative and primed states, as well as to down-regulate Nanog in the formative state. Overall, *Pou5f1*^∆/∆^;*Rosa26^Pou5f1/+^* cells failed to properly enter these states of pluripotency on their way to terminal differentiation.

It is likely that observed defects of *Pou5f1^∆/∆^;Rosa26^Pou5f1/+^* cells are caused due to an improper regulation of *Pou5f1* translocated to the *Rosa26* locus. Although DE was previously defined DE as crucial for *Pou5f1* expression in the naïve state, there may be additional regulatory elements that maintain *Pou5f1* expression at an appropriate level. Of note, recently described *Pou5f1* elements may serve this purpose [16]. Mouse orthologs should be examined for their ability to regulate *Pou5f1* expression as well. These elements were revealed in human ESCs, which were in the primed pluripotency state, although *Pou5f1^∆/∆^;Rosa26^Pou5f1/+^* cells exhibited the most severe abnormalities during the transition from naïve to primed pluripotency states. Furthermore, under the naïve conditions, Oct4 expression in *Pou5f1^∆/∆^;Rosa26^Pou5f1/+^* ESCs nearly caught up with that in *Pou5f1^flox/∆^* cells, while the elements revealed by Diao et al. acted as enhancers, and their lack led to a reduced Oct4 expression. More relevant to our case, the deletion of an element just downstream of the *Pou5f1* RNA-coding sequence, which is not considered critical for *Pou5f1* regulation (contrary to the DE and PE), led to approximately 50% up-regulation of randomly integrated *Pou5f1(GOF)-lacZ* transgene in the inner cell mass of preimplantation embryos, which contain epiblast cells in the naïve state of the pluripotency [11]. The authors did not assess the impact of this element in the context of a naïve-to-primed state transition, but the *Rosa26^Pou5f1^* allele in our cells also lacked this downstream element, which might be the main reason for the observed phenotype. An alternative possibility is that the observed mis-regulation was caused by genetic elements located in the *Rosa26* locus. In this case, these putative elements should act selectively upon 9.8 kb *Pou5f1* transgene as they do not affect the expression of CAG- transgene upon S/L to 2i/L condition switch. Further experiments using *Pou5f1* translocation into other safe-harbor or random genomic loci should address the issue of *Pou5f1* transgene–interaction with the genetic surrounding.

Another observation is rather intriguing. Though *Pou5f1^∆/∆^*;*Rosa26^Pou5f1/+^* cells after 9–11 days of culturing under 2i/L conditions were close to the control cells in terms of naïve state markers expression (Oct4, Nanog, and Klf4) and showed no sign of premature transition to the formative/primed state (Oct6), prolonged exposure to the 2i/L conditions partially restored cell proliferation and survival of *Pou5f1^∆/∆^*;*Rosa26^Pou5f1/+^* ESCs (Figure 3 and Figure S2). This means that these ESCs can adapt to *Pou5f1* mis-regulation via mechanisms such as epigenetic erasing, which is a known feature of the naïve pluripotency state [25,26,27].

Overall, our data suggest that correct spatiotemporal regulation of the *Pou5f1* gene requires *cis*-regulatory elements in addition to the previously characterized DE and PE [11,12]. Future attempts should focus on identifying these elements, and we believe that our approach, combined with modern techniques of chromatin state assessment, will be of great help in this effort.

## 4. Materials and Methods

### 4.1. Animals

All animal procedures were performed according to the guidelines for the humane use of laboratory animals, using standards corresponding to those prescribed by the American Physiological Society. The derivation of mouse embryonic fibroblast (MEFs) and teratoma formation was performed in the Institute of Cytology strictly in agreement with the animal protection legislation acts of the Russian Federation and was approved as humane use of laboratory animals by the Institute’s Ethical Board (protocol No. 17/22, signed 9 November 2022).

### 4.2. Cell Culture

All cells were cultured in humidified CO_2_ incubators at 20% O_2_ and 5% CO_2_ at 37 °C. MEFs derived from CBA embryos as per the established procedure [28] were routinely cultured on gelatin-coated cell culture dishes in DMEM GlutaMAX (Gibco, ThermoFischer Scientific, Waltham, MA USA) supplemented with 10% FBS (Biosera, Cholet, France) and 1 × penicillin/streptomycin (Gibco) passaged using 0.05% Trypsin-0.01% EDTA (Gibco). On passage 3–5, MEFs were treated for 2.5 h with 10 μg/mL mitomycin C (MMC; Sigma-Aldrich M0503, Darmstadt, Germany). Mouse ESCs were cultured on the MMC-inactivated MEFs (pre-seeded at a density of approximately 30,000 cells/cm^2^ on gelatin-coated cell culture dishes) in KnockOut DMEM (Gibco) supplemented with 15% FBS (Biosera), 1× NEAA (Gibco), 1× penicillin/streptomycin (Gibco), 0.1 mM 2-mercaptoethanol (Sigma-Aldrich), 2 mM l-glutamine (Gibco), and 1:5000 of in-house-made hLIF, referred to as S/L medium. ESCs were put into naïve pluripotency state by culturing on poly-L-ornithine (0.01%; Sigma)-coated plates in the N2B27 media supplemented with, 3 μM CHIR99021 (Axon, Groningen, The Netherlands), 1 μM PD0325901 (Axon), and 1:5000 of in-house made hLIF, designated as the 2i/L medium. The N2B27 media included DMEM/F12 (Gibco) and Neurobasal medium (1:1), supplemented with 1× N2, 1× B27 (without RA) (Gibco), 50 μM β-mercaptoethanol (Sigma Aldrich), 0.005% BSA (Sigma), 1× penicillin/streptomycin (Gibco), and 2 mM L-glutamine (Gibco). To promote differentiation into EpiLCs, ESCs cultured in the 2i/L medium were plated for 2 days on fibronectin-coated plates in N2B27 media supplemented with 1% knockout serum replacement (KSR; ThermoFisher Scientific, Waltham, MA USA), 20 ng/mL activin, and 12 ng/mL bFGF. To further proceed to the differentiation into EpiSCs, media was additionally supplemented with 6.25 mg/mL XAV 939.

### 4.3. Transient Transfection

ESCs were seeded at a density of 5 × 10^3^/cm^2^ per well of 24-well plates. The next day, media were changed to fresh S/L media. After two hours, the transfection mix (1:2 ratio, mixed in 250 µL of OptiMEM) was added into the wells. Twelve hours post-transfection, the media were changed to the fresh S/L medium; after an additional 24 h, medium was replaced with the same medium containing antibiotics.

### 4.4. Establishment of Mutant ESC Lines

Derivation of *Pou5f1^∆/∆^;Rosa26^Pou5f1/+^* ESCs was previously reported [17]. To generate *Pou5f1^∆/flox^* ESCs, ESCs derived from *Pou5f1^flox/flox^*, blastocysts were seeded at a density of 5 × 10^3^ per cm^2^ and, with the purpose to achieve partial recombination, transiently transfected with the Cre-expressing plasmid *pRosa26-TRE-CAG-Frt(Ert2CreErt2-STOP)Frt-tdTomatoPGKneo* [10,24]. Twenty-four hours after transfection, the media were changed to the S/L media containing 4-hydroxytamoxifen (4-OHT). The next day, cells were seeded in a 6-well plate and cultured in the presence of 500 µg/mL Geneticin (G-418) (#108321-42-2, bioWORLD, Dublin, Ohio, USA) for 4 days.

### 4.5. Immunocytochemistry

Cells were fixed in 4% PFA (Sigma) for 10 min, permeabilized with 0.1% Triton X-100 for 15 min, blocked in 3% BSA for 1 h at RT, and incubated with primary antibodies overnight at 4 °C. The next day, cells were washed 5–6 times with PBS, incubated with appropriate secondary fluorescent antibodies for 2 h at RT, and then washed and stained with DAPI. Immunochemistry images were taken on the fluorescent microscope EVOS FL AUTO (Life Technologies, ThermoFischer Scientific, Waltham, MA USA). The following primary antibodies were used: mouse anti-Oct4 (1:500, C-10, sc-5279, Santa Cruz Biotechnology, Dallas, TX, USA), rabbit anti-Oct4 (1:300, ab19857, Abcam, Cambridge, UK), rat anti-Nanog (1:250, 14-5761-80, eBioscience, ThermoFischer Scientific, Waltham, MA USA), rabbit anti-Klf4 (1:250, ab129473, Abcam), and rabbit anti-Oct6 (1:250, ab272925, Abcam). The following secondary antibodies were used at 1:500 dilutions: goat-anti-rat Alexa Fluor 555 (Invitrogen, ThermoFischer Scientific, Waltham, MA USA), goat-anti-mouse Alexa Fluor 488, and goat-anti-rabbit Alexa Fluor 647 (Jackson ImmunoResearch, West Grove, PA, USA).

### 4.6. Annexin V-FITC/PI Staining

Annexin V and PI staining were performed using the Annexin V-FITC Apoptosis Detection Kit (ab14085, Abcam) according to the manufacturer’s instructions. In brief, cells were washed with PBS and harvested at 300 g for 3 min; the supernatant was discarded, and the pellet was resuspended in 1× binding buffer at a density of 1–5 × 10^5^ cells per mL. Samples were then incubated with 5 μL of FITC-conjugated annexin V for 15 min at RT in a dark place. Propidium iodide (PI) was added to the samples right before analysis. Flow cytometry was performed using the CytoFLEX flow cytometer (Beckman Coulter, Brea, CA, USA), and data were analyzed using CytoFLEX CytExpert software (ver 2.3).

### 4.7. Teratoma Formation Assay

Mouse ESCs, cultured on gelatin-coated dishes in ESC medium, were harvested with 0.05% Trypsin-0.01% EDTA (Gibco), resuspended in PBS, and injected subcutaneously (2 × 10^6^ cells) into athymic CD-1 NUDE mice maintained under specific pathogen-free conditions. Four animals were used, with two injections per mouse. Five weeks after the injections, the mice were sacrificed by cervical dislocation and the teratomas were surgically removed. We then proceeded to sectioning and histological analysis.

### 4.8. Preparation of Sections for Histological Analysis

Excised teratomas were washed in PBS and fixed for 24 h at RT in two types of fixators: Bouin’s fluid and 10% formalin. After performing Bouin’s fixation, the teratomas were washed in several changes of 70% alcohol until the solution was lightened. After the formalin fixation, teratomas were washed in running water for 10 min and transferred to 70% alcohol. Specimens were dehydrated in an ethanol series and isobutanol:paraffin series and then embedded in paraffin (McCormick Scientific, now Leica Biosystems, Nussloch, Germany). For each teratoma, blocks of approximately 5 × 5 mm were used for analysis. Paraffin sections were washed in xylene, rehydrated using an ethanol series (100–70%), and then washed with water. Next, sections were incubated in hematoxylin for 6 min, washed with water, and incubated with eosin for 3 min. After washing and dehydration, sections were mounted in Vitrogel (BioVitrum, St-Petersburg, Russia) for further analysis.

### 4.9. Western Blotting

We lysed 1 × 10^6^ cells in the RIPA buffer (Invitrogen) containing cOmplete™ ULTRA protease-inhibitor (Roche, Basel, Switzerland) and sonicated the products. SDS-PAGE electrophoresis was performed using 10% polyacrylamide gel in a Tris-HCl buffer. Protein transfer was performed using the CD10 semi-dry system (Cleaver Scientific, Rugby, UK). After blocking with 3% BSA, the membranes were incubated with the following primary antibodies: mouse anti-Oct4 (1:1000, C-10, sc-5279, Santa Cruz Biotechnology), rabbit anti-Oct4 (1:1000, ab19857, Bethyl, Fortis Life Sciences, Waltham, MA USA), rabbit anti-GAPDH (1:2000, 2118s, Cell signaling, Danvers, MA, USA). After washing with PBS, membranes were incubated with HRP-conjugated secondary antibodies (1:10,000, Jackson ImmunoResearch, Ely, UK). Visualization was performed using a Chemidoc Touch device (BIO-RAD, Hercules, CA, US).

### 4.10. Quantification and Statistical Analysis

To determine cellular proliferation, all cell lines were seeded with the same cell concentration and monitored by flow cytometry every 3–4 days. The number of cells in control (*Pou5f1^flox/flox^*) ESCs was taken as 100%. Four biological replicates were used for each cell line to calculate growth rates and apoptosis levels under the S/L and 2iL conditions. Three biological replicates were employed to evaluate the relative intensity of the band. IMAGEJ Fiji was utilized to carry out the analysis of Western blot images. The data is presented as mean ± SEM (Standard error of the mean). The statistical significance of small samples was evaluated using the non-parametric Mann-Whitney U-test. Differences were considered statistically significant at *p* < 0.05.

## Data Availability

The datasets used and/or analyzed during the current study are available from the corresponding author upon reasonable request.

## Supplementary Materials

The following supporting information can be downloaded at: https://www.mdpi.com/article/10.3390/ijms242015434/s1.
