# Supplementary material for: Regulatory Elements Outside Established Pou5f1 Gene Boundaries Are Required for Multilineage Differentiation of Embryonic Stem Cells
Source: Int J Mol Sci. 2023 Oct 21;24(20):15434. doi: 10.3390/ijms242015434 (PMC10607089; doi:10.3390/ijms242015434)
Supplement: Supplementary file 1 [file ijms-24-15434-s001.zip › ijms-2575293-supplementary.pptx]

## Slide 1
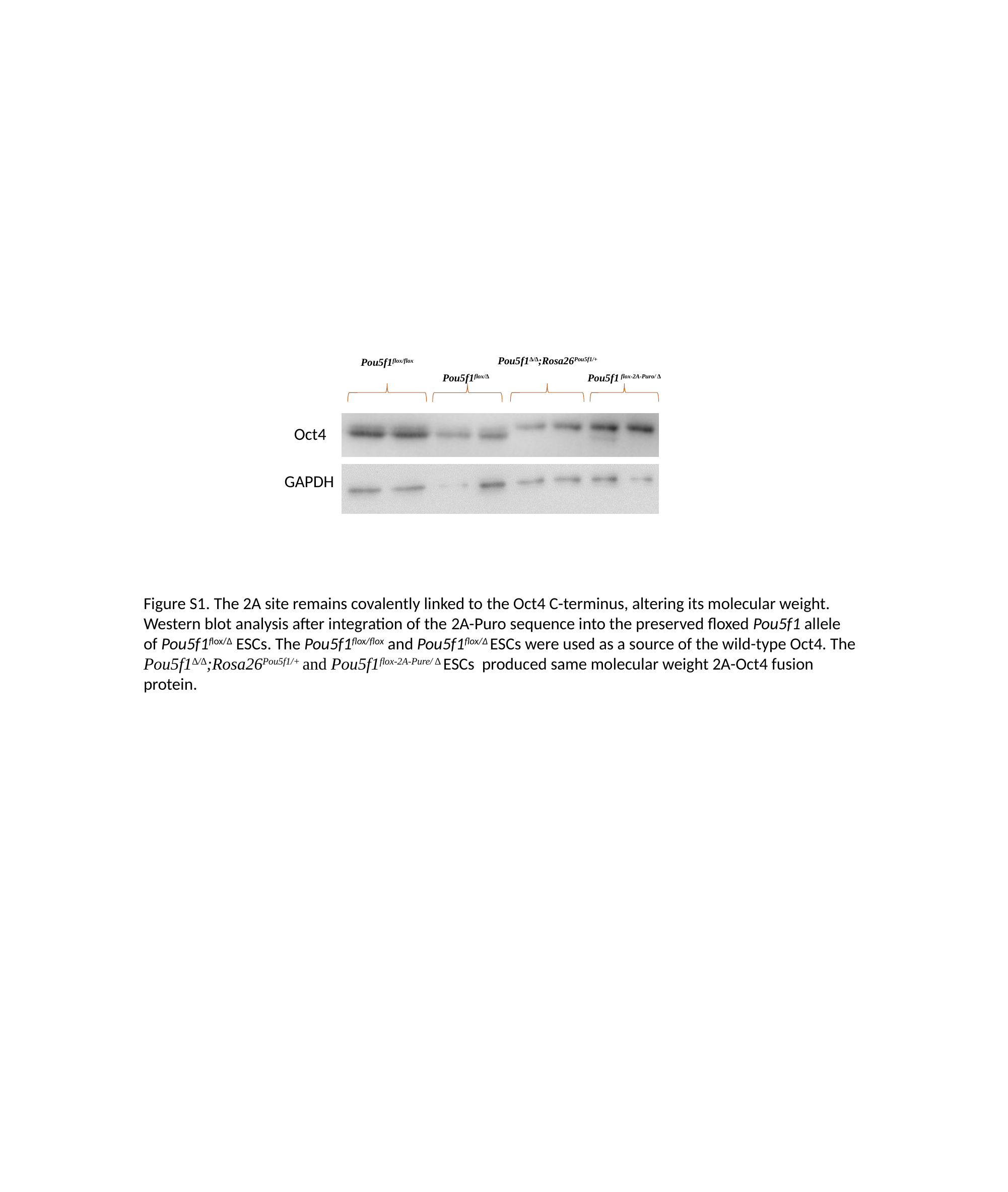

Pou5f1∆/∆;Rosa26Pou5f1/+
Pou5f1flox/flox
Pou5f1 flox-2A-Puro/ ∆
Pou5f1flox/∆
Oct4
GAPDH
Figure S1. The 2A site remains covalently linked to the Oct4 C-terminus, altering its molecular weight. Western blot analysis after integration of the 2A-Puro sequence into the preserved floxed Pou5f1 allele of Pou5f1flox/∆ ESCs. The Pou5f1flox/flox and Pou5f1flox/∆ ESCs were used as a source of the wild-type Oct4. The Pou5f1∆/∆;Rosa26Pou5f1/+ and Pou5f1flox-2A-Pure/ ∆ ESCs produced same molecular weight 2A-Oct4 fusion protein.

## Slide 2
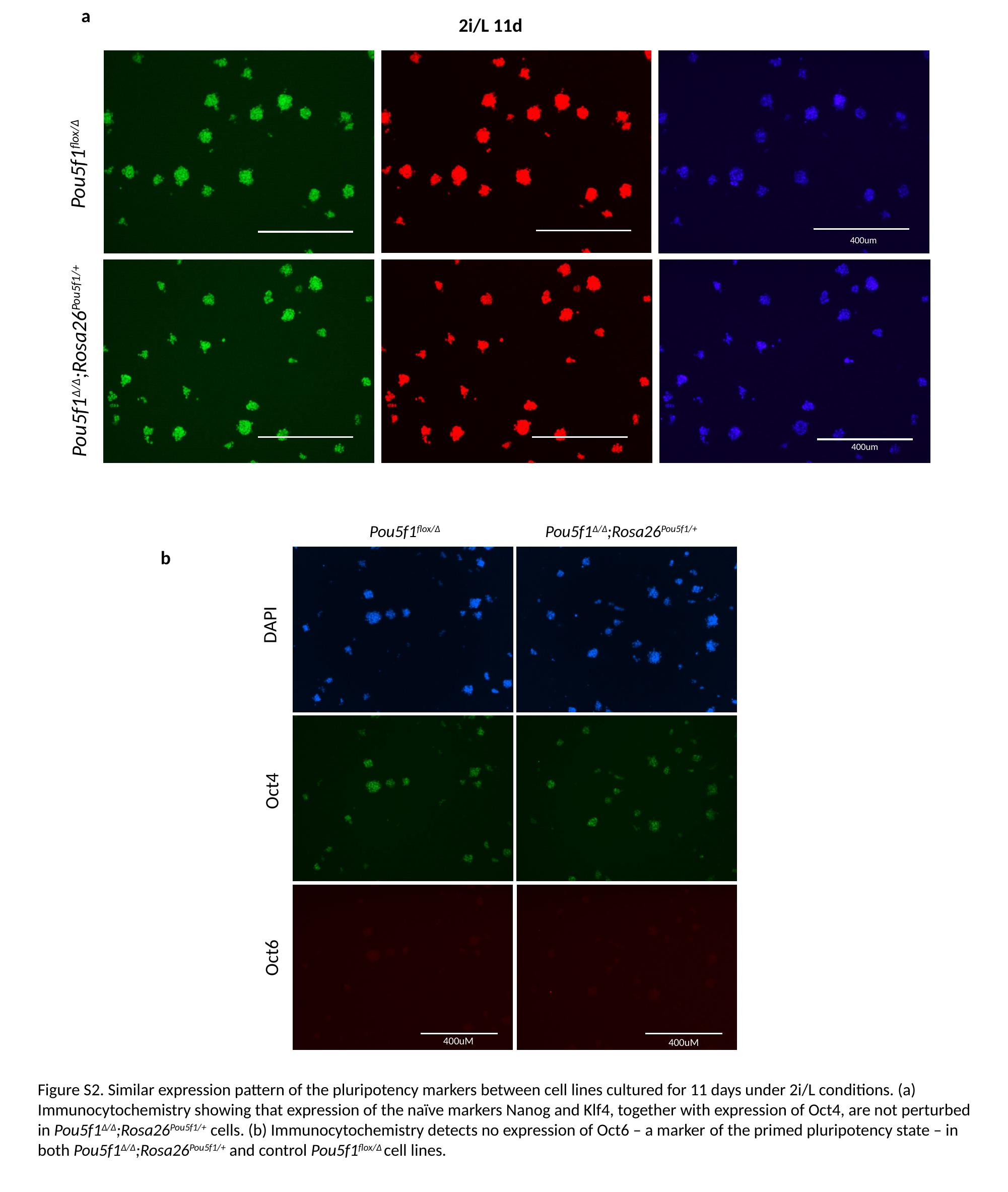

a
2i/L 11d
Pou5f1flox/∆
400um
Oct4
Klf4
Nanog
Pou5f1∆/∆;Rosa26Pou5f1/+
400um
Pou5f1flox/∆
Pou5f1∆/∆;Rosa26Pou5f1/+
b
DAPI
Oct4
Oct6
400uM
400uM
Figure S2. Similar expression pattern of the pluripotency markers between cell lines cultured for 11 days under 2i/L conditions. (a) Immunocytochemistry showing that expression of the naïve markers Nanog and Klf4, together with expression of Oct4, are not perturbed in Pou5f1∆/∆;Rosa26Pou5f1/+ cells. (b) Immunocytochemistry detects no expression of Oct6 – a marker of the primed pluripotency state – in both Pou5f1∆/∆;Rosa26Pou5f1/+ and control Pou5f1flox/∆ cell lines.

## Slide 3
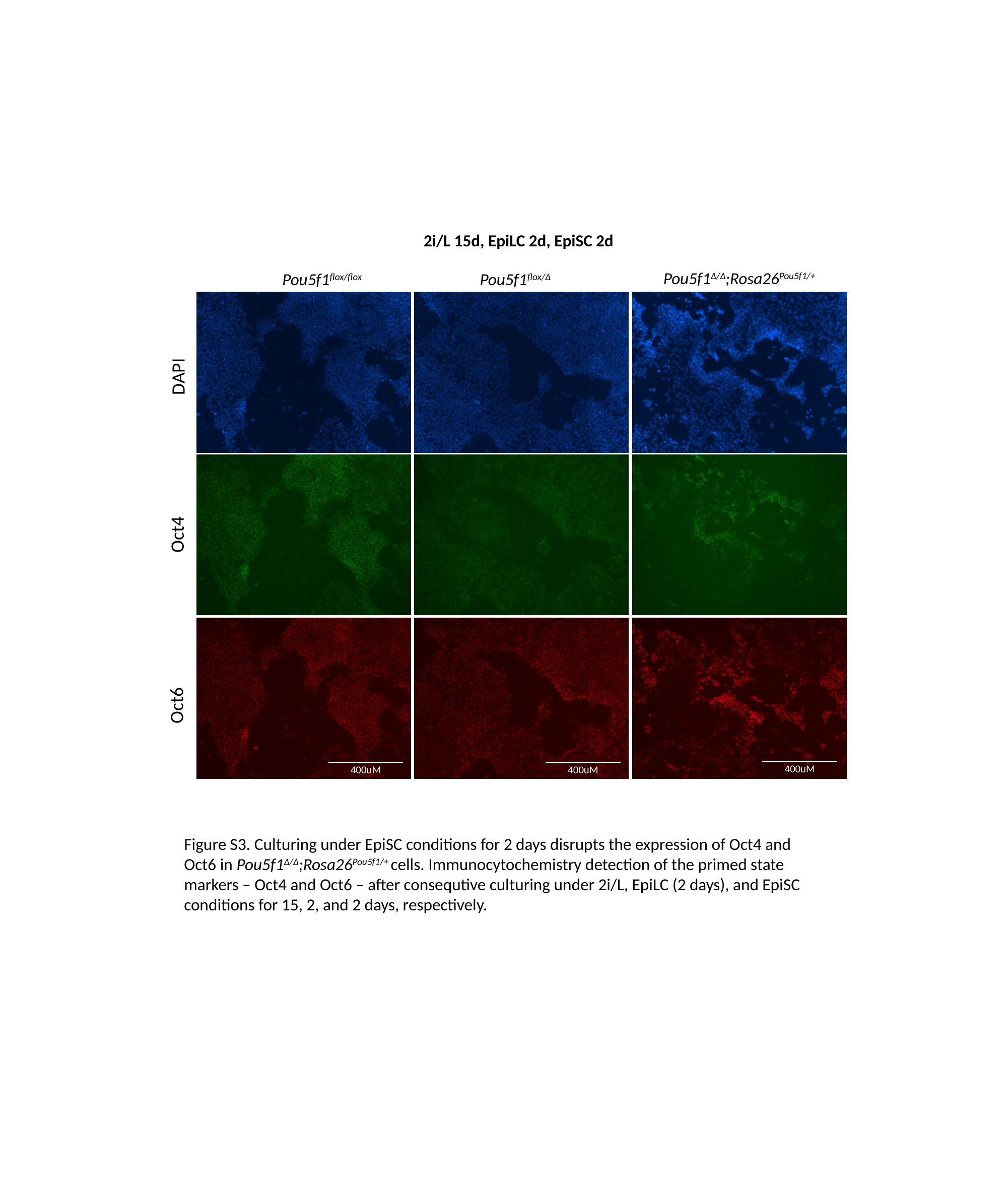

2i/L 15d, EpiLC 2d, EpiSC 2d
Pou5f1∆/∆;Rosa26Pou5f1/+
Pou5f1flox/flox
Pou5f1flox/∆
DAPI
Oct4
Oct6
400uM
400uM
400uM
Figure S3. Culturing under EpiSC conditions for 2 days disrupts the expression of Oct4 and Oct6 in Pou5f1∆/∆;Rosa26Pou5f1/+ cells. Immunocytochemistry detection of the primed state markers – Oct4 and Oct6 – after consequtive culturing under 2i/L, EpiLC (2 days), and EpiSC conditions for 15, 2, and 2 days, respectively.

## Slide 4
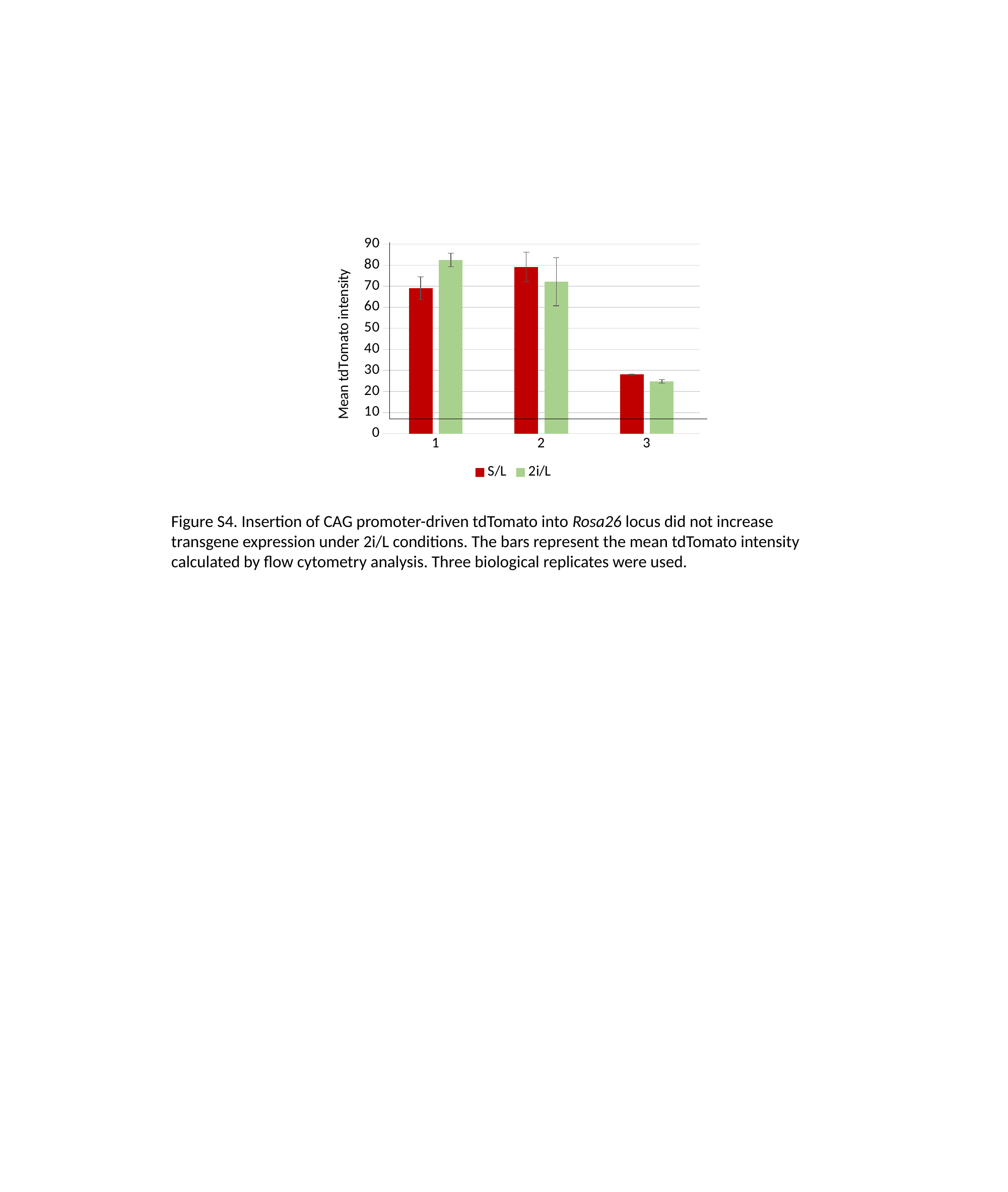

### Chart
| Category | S/L | 2i/L |
|---|---|---|Figure S4. Insertion of CAG promoter-driven tdTomato into Rosa26 locus did not increase transgene expression under 2i/L conditions. The bars represent the mean tdTomato intensity calculated by flow cytometry analysis. Three biological replicates were used.

## Slide 5
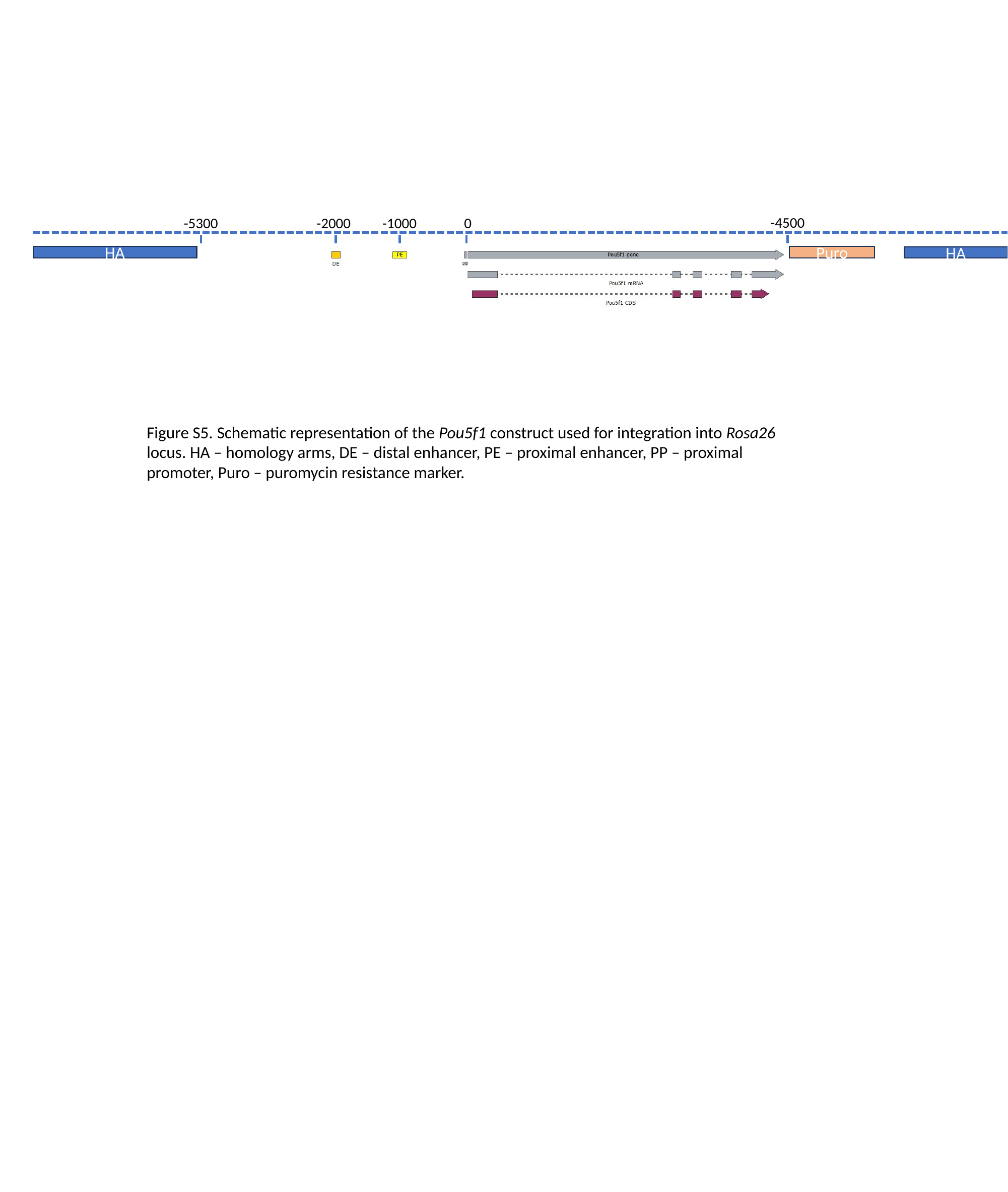

-4500
-5300
-2000
-1000
0
Puro
HA
HA
Figure S5. Schematic representation of the Pou5f1 construct used for integration into Rosa26 locus. HA – homology arms, DE – distal enhancer, PE – proximal enhancer, PP – proximal promoter, Puro – puromycin resistance marker.
